# Dasatinib as the salvage therapy for chronic myeloid leukemia with blast crisis and central nervous system involvement: A case report

**DOI:** 10.3892/ol.2015.2928

**Published:** 2015-02-03

**Authors:** SHIUE-WEI LAI, TZU-CHUAN HUANG, JIA-HONG CHEN, YI-YING WU, PING-YING CHANG

**Affiliations:** Department of Internal Medicine, Division of Hematology and Oncology, Tri-Service General Hospital, National Defense Medical Center, Taipei, Taiwan, R.O.C.

**Keywords:** dasatinib, chronic myeloid leukemia, blast crisis, central nervous system

## Abstract

BCR-ABL tyrosine-kinase inhibitors are the first-line therapy for the majority of patients with chronic myelogenous leukemia (CML). Up to 20% of patients who have imatinib-treated CML in blast crisis (BC) experience a relapse in the central nervous system (CNS) due to the poor penetration of the drug by the blood-brain barrier. The present case reports a successful experience of using dasatinib-based combination therapy to treat a 22-year-old female who presented with initial symptoms of intermittent fever and easy bruising under the diagnosis of CML in BC. Although the patient eventually succumbed to profound sepsis, the CNS involvement was treated successfully using dasatinib-based combination therapy (cranial radiation and de-escalated intrathecal chemotherapy). This case demonstrates that dasatinib may be a viable option for those who are not medically fit for or are otherwise unwilling to receive high-dose chemotherapy. It appears that dose intensity is essential for optimal efficacy and should be maintained at 150 mg daily as far as possible.

## Introduction

Chronic myeloid leukemia (CML) is a myeloproliferative disorder that is associated with a unique translocation of chromosomes 9 and 22, resulting in the so-called Philadelphia chromosome (Ph). This in turn results in the formation of a fusion protein product, BCR-ABL, which plays a key role in the pathogenesis of CML. Imatinib is the first-generation tyrosine kinase inhibitor (TKI) of BCR-ABL and is approved to treat Ph-positive (Ph^+^) leukemia (1). Although the drug is highly active, it is noteworthy that 30–35% of patients do not have an optimal response due to treatment interruptions resulting from intolerance to the adverse effects (2).

Up to 20% of patients who have imatinib-treated CML in blast crisis (BC) or Ph^+^ acute leukemia experience a relapse in the central nervous system (CNS) (3,4). This is a challenging issue in the imatinib era, and is due to the poor penetration of the drug by the blood-brain barrier (BBB) (4–10). Dasatinib and nilotinib, the second-generation TKIs, lead to a complete cytogenetic response (CyR) in ~50% of imatinib-resistant patients and to major molecular remission in 20–30% (11,12). Furthermore, the two TKIs have demonstrated hematological and molecular responses higher than those of imatinib, and are approved for first-line treatment of newly diagnosed CML in the chronic phase (CP) (13,14). At least one previous study has suggested that dasatinib crosses the BBB and is effective for CNS Ph^+^ leukemia (15). However, other studies have reported conflicting findings (16–21). The present study reports a successful experience of using dasatinib-based combination therapy to treat CNS involvement in a 22-year-old female with CML in BC. Written informed consent was obtained from the patient’s family.

## Case report

**Figure 1:**
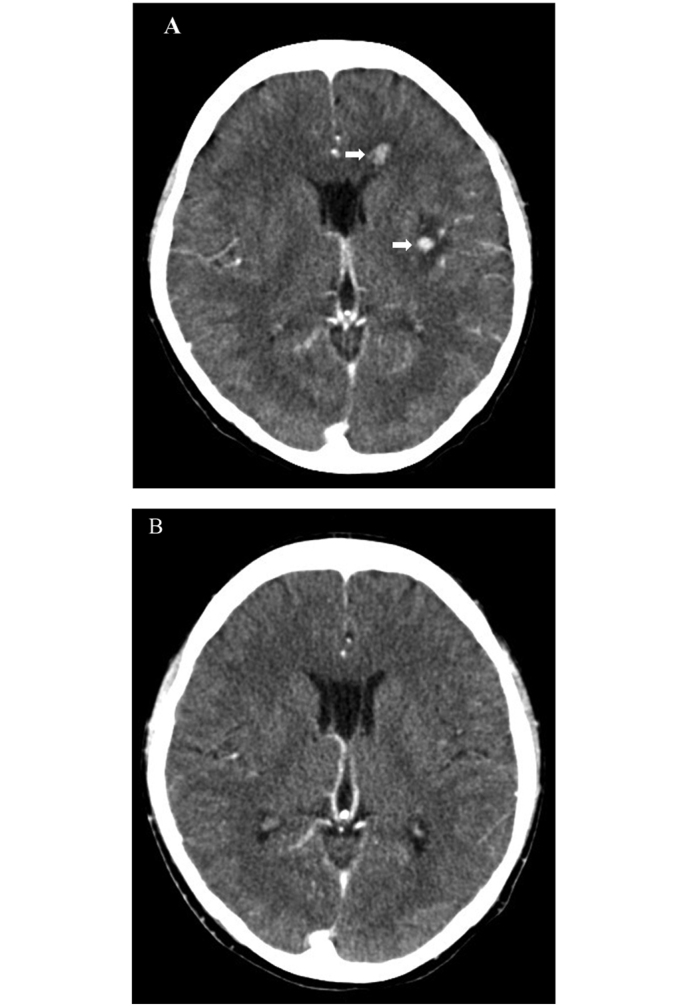
Computed tomography scan of the brain. (A) Multiple small enhancing nodules (white arrows), with mild perifocal edema in the bilateral cerebrum and left cerebellum. (B) Normal gray and white matter attenuation without evidence of abnormal enhancing lesions, demonstrated following dasatinib treatment and whole brain radiotherapy.

A 22-year-old female was diagnosed with CML in CP at Tri-Service General Hospital (Taipei, Taiwan) in November 2008 and was treated with imatinib 400 mg daily. The patient showed poor compliance with frequent drug interruptions, and the best response during treatment was a major CyR. In October 2012, approximately four years after the initiation of therapy, the patient experienced a new onset of intermittent fever and easy bruising. Laboratory testing revealed marked leukocytosis, with a white blood cell (WBC) count of 330,000 μl (normal range, 4,500–11,000 μl) and excessive peripheral myeloblasts (53.4%; normal range, 0–1%). A subsequent bone marrow (BM) biopsy revealed 60% myeloblasts (normal range, 0.3–5%), and a diagnosis of CML in BC was made. No mutation was detected in the leukemic BCR-ABL transcripts. The patient received induction chemotherapy with seven days of 160 mg cytarabine (100 mg/m^2^) and three days of 100 mg daunorubicin (60 mg/m^2^). A repeat BM examination revealed partial remission after two weeks. Due to a new onset of left facial numbness, the patient underwent a computed tomography (CT) scan of the brain, which revealed multiple small enhancing nodules (Fig. 1A). Cerebrospinal fluid (CSF) examination revealed myeloperoxidase-positive blasts.

Due to a poor Eastern Cooperative Oncology Group performance status of 3, it was decided not to treat the patient with aggressive systemic or intrathecal therapy at that time. Instead, the patient was treated with 50 mg dasatinib three times a day in combination with whole brain radiotherapy (total, 3,000 cGy). A repeat CT scan revealed near-complete resolution of the brain lesions (Fig. 1B). A repeat BM examination also revealed complete remission. During the treatment, only grade 1 hematological toxicities were noted. The patient was subsequently discharged and kept on the same dose of dasatinib.

**Figure 2:**
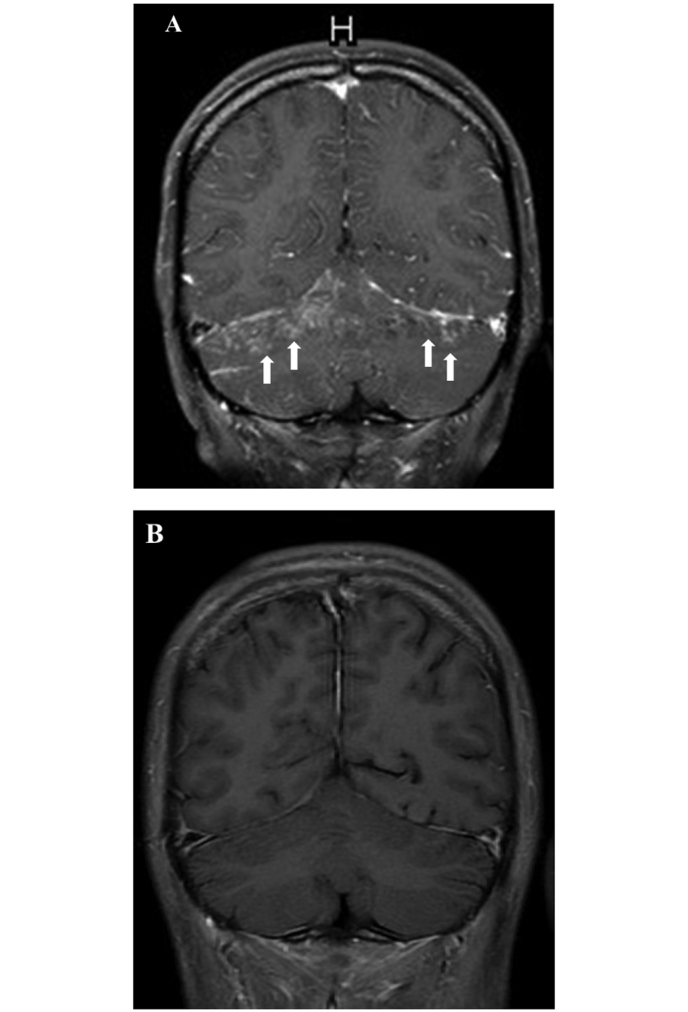
Gadolinium diethylenetriaminepentacetate-enhanced coronal T1-weighted image of the brain. (A) Diffuse leptomeningeal enhancement (white arrows) in the sulci of the bilateral cerebri and cerebelli. (B) Significant regression of the leptomeningeal enhancement, observed following treatment with dasatinib and intrathecal chemotherapy.

**Figure 3:**
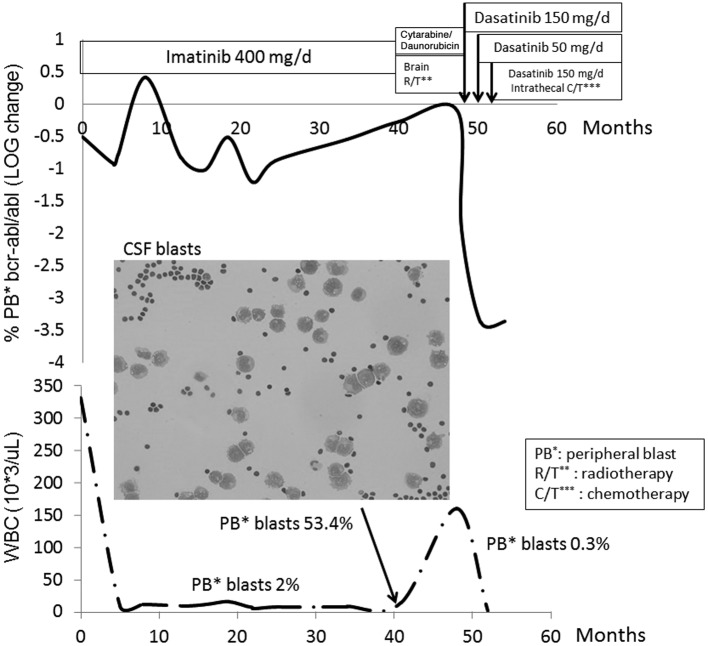
Clinical course of the present patient with Philadelphia chromosome-positive chronic myelogenous leukemia and recurrent central nervous system relapse while undergoing dasatinib combination therapy (CSF blast image; stain, Diff-Quik; magnification, ×400). CSF, cerebrospinal fluid; WBC, white blood cell.

One month later, the patient developed intermittent vomiting and a fever. Laboratory testing revealed pancytopenia, with a WBC count of 2,590 μl (normal range, 4,500–11,000 μl), a hemoglobin concentration of 8.4 g/dl (normal range, 11.3–15.3 g/dl) and a platelet count of 109,000 μl (normal range, 150,000–400,000 μl). Since subsequent investigation did not reveal any notable cause, this was suspected to be due to the dasatinib treatment and so the dose was reduced. The next week, three days after the dose reduction of dasatinib, the patient experienced a generalized tonic-clonic seizure. Magnetic resonance imaging (MRI) of the brain revealed diffuse leptomeningeal enhancement (Fig. 2A). The patient became rapidly obtunded and required intubation for ventilator support. Following weekly intrathecal chemotherapy [methotrexate (12 mg), cytarabine (100 mg) and hydrocortisone (50 mg)] for five courses, in combination with 50 mg dasatinib three times daily, the patient’s clinical condition gradually improved. Subsequently, the patient was successfully weaned off the ventilator and discharged. Furthermore, a repeat brain MRI revealed significant regression of the brain lesions (Fig. 2B). Grade 3 hematological toxicities were noted during the treatment. Two weeks after being discharged, the patient experienced confusion and generalized weakness. A brain MRI revealed diffuse leukoencephalopathy, but there was no evidence of CNS relapse on a repeat CSF examination. The patient was admitted to the hospital, but succumbed to pneumonia with profound sepsis two weeks after admission. The entire treatment course in correlation with BCR-ABL transcript levels is shown in Fig. 3.

## Discussion

Acute leukemia with CNS involvement is not uncommon in clinical practice. Intrathecal chemotherapy, high-dose chemotherapy and radiotherapy are conventional treatments for CNS leukemia. However, a poor quality of life, significant systemic or CNS toxicity, a short response duration and ultimately mortality associated with refractory leukemia are common outcomes in a number of patients (22).

Imatinib is a substrate for the drug-eluting P-glycoprotein, which results in suboptimal penetration into the CNS, and the CSF levels of imatinib are 100-fold less than those achieved in plasma (6–8). This may explain why patients have CNS relapse in spite of achieving a complete response in the peripheral blood and BM. Dasatinib has greater potency (325-fold) than imatinib and can be therapeutically effective at a low or even subnanomolar concentration (23,24). A study by Porkka *et al* demonstrated that dasatinib is associated with substantial clinical responses in patients with CNS leukemia and could significantly increase survival and control intracranial tumors *in vivo* (15). In addition to the present study, four separate case reports in Table I further support the potential benefit of dasatinib in Ph^+^ CNS leukemia (16–19). In these four cases, the majority of patients received dasatinib combination therapies and all patients were administered ≥140 mg dasatinib, daily (16–19). Nishimoto *et al* reported that dasatinib maintenance following allogeneic hematopoietic stem cell transplantation has the potential to prevent CNS relapse (18). In spite of these encouraging studies, it is sobering that several patients have progressive disease within months of starting therapy. Notably, Papageorgiou *et al* reported one case of Ph^+^ acute megakaryoblastic leukemia who received 140 mg dasatinib daily and maintained stable disease for 16 months, however, the patient experienced CNS relapse following treatment with a de-escalated daily dose of 70 mg daily due to pleural effusion (20). Frigeri *et al* also presented a case of Ph^+^ CNS leukemia in which dasatinib failed to prevent CNS progression. However, this patient was administered <100 mg dasatinib daily during the treatment course (21).

While disease biology may play a significant role, it is vital to investigate whether other factors may be involved. One possibility may be the loss of CNS disease control with the lowering of the dasatinib dose. Indeed, it appears that among the cases reported in the literature, outcomes are poor when the dose is <140 mg a day (15–21). The most common reasons for decreasing the dose of dasatinib are adverse events, including cytopenia or pleural effusion (25). This was also observed in the patient in the present study, where progressive neurological deterioration occurred shortly after dasatinib dose reduction and a marked improvement was noted following re-escalation to 150 mg once daily (Fig. 2). Although the overall experience with this issue is limited to the small number of cases in the literature, we believe that the available anecdotal data point to a requirement for a sufficient dose intensity of dasatinib for improved outcomes.

In conclusion, dasatinib may be a viable option for the management of patients with Ph^+^ CNS leukemia, including those who are not medically fit for or are otherwise unwilling to receive high-dose chemotherapy. It appears that dose intensity is essential for optimal efficacy and should possibly be maintained at 150 mg daily as far as possible. A well-designed, prospective study will aid in further clarifying this issue and better defining the role of dasatinib in this setting.

## Figures and Tables

**Table I:** DA combination therapies for PH^+^ CNS leukemia.

Ref.	Patient	BCR/ABL mutation	Prior HSCT	Combination therapies	DA dosage, mg/day	DA duration after CNS leukemia	Best CNS response	Alive	Treatment and outcome
16	Ph^+^ AL^a^	T315I	Yes	IT	140	52 days	PR	No	200 mg/day since day 37; succumbed to disease progression.
17	BC-CML	T315I^b^	No	IT	140	4 months	CR	Yes	Awaiting HSCT
18	BC-CML	NR	No	RT, IT	140	38+ months	CR	Yes	Post-HSCT DA maintenance; leukoencephalopathy
19	Ph^+^ ALL	NR	Yes	RT, IT	140	12 months	CR	Yes	
20	Ph^+^ AML	No	No	No	70	7 months	PD	No	Initially 140 mg/day, 16 months, then 70 mg/day, 7 months, due to pleural effusion; succumbed to CNS relapse
21	BC-CML	No	Yes	IT	100	4 months	PD	No	Succumbed to CNS relapse
Present case	BC-CML	No	No	RT, IT	150	6 months	CR	No	Leukoencephalopathy; succumbed to pneumonia with sepsis

DA, dasatinib; CNS, central nervous system; Ph^+^, Philadelphia chromosome-positive; BC-CML, chronic myeloid leukemia blast crisis; ALL, acute lymphoblastic leukemia; AML, acute megakaryoblastic leukemia; RT, radiotherapy; IT, intrathecal chemotherapy; SCT, stem cell transplantation; NR, not reported; CR, complete remission; PD, progressive disease; HSCT, hematopoietic stem cell transplantation.

a Ph^+^ biphenotypic acute leukemia; relapse of leukemia with T315I mutation on day 27.

b After dasatinib treatment for 2 months.
